# Using continuous data on tumour measurements to improve inference in phase II cancer studies

**DOI:** 10.1002/sim.5867

**Published:** 2013-06-18

**Authors:** James MS Wason, Shaun R Seaman

**Affiliations:** MRC Biostatistics UnitCambridge, U.K.

**Keywords:** continuous tumour shrinkage endpoints, informative dropout, longitudinal model, phase II cancer trial

## Abstract

In phase II cancer trials, tumour response is either the primary or an important secondary endpoint. Tumour response is a binary composite endpoint determined, according to the Response Evaluation Criteria in Solid Tumors, by (1) whether the percentage change in tumour size is greater than a prescribed threshold and (2) (binary) criteria such as whether a patient develops new lesions. Further binary criteria, such as death or serious toxicity, may be added to these criteria. The probability of tumour response (i.e. ‘success’ on the composite endpoint) would usually be estimated simply as the proportion of successes among patients. This approach uses the tumour size variable only through a discretised form, namely whether or not it is above the threshold. In this article, we propose a method that also estimates the probability of success but that gains precision by using the information on the undiscretised (i.e. continuous) tumour size variable. This approach can also be used to increase the power to detect a difference between the probabilities of success under two different treatments in a comparative trial. We demonstrate these increases in precision and power using simulated data. We also apply the method to real data from a phase II cancer trial and show that it results in a considerably narrower confidence interval for the probability of tumour response.

## 1.Introduction

Phase II cancer trials are conducted to decide whether an experimental cancer treatment is worth testing in a large, costly phase III trial. Traditionally, cancer agents were cytotoxic, that is, designed to destroy tumour cells, and phase II cancer trials were single-arm trials that compared the anti-tumour activity of the experimental drug with historical control data 1. For cytotoxic drugs, tumour shrinkage remains a widely used primary endpoint. This is because a cytotoxic agent would have to display some level of anti-tumour activity in order to have a positive effect on overall survival, the usual primary endpoint in phase III cancer trials. In recent times, cytostatic drugs have become increasingly common. Cytostatic drugs are molecularly targeted agents that are designed to improve survival through mechanisms other than directly destroying tumour cells and so in phase II trials are primarily assessed through progression-free survival 2. However, whether the tumour increases in size is often an important secondary outcome. This is because if the agent fails to control tumour growth, survival is likely to be shortened. Thus, in phase II trials of both cytotoxic and cytostatic drugs, change in the size of the tumour is an important outcome. Although phase II cancer trials were traditionally single-arm trials, in recent times, randomised trials have become more common.

The most common way of assessing change in size of the tumour is the Response Evaluation Criteria in Solid Tumors (RECIST) 3. RECIST classifies patients into complete responses (CR), partial responses (PR), stable disease (SD) or progressive disease (PD). Generally, in trials of cytotoxic agents, CR and PR are classed as treatment successes, with SD and PD classed as treatment failures. The proportion of patients that are PR or CR is called the objective response rate (ORR). In trials of cytostatic agents, SD is included in treatment success, and the proportion of patients that are successful is called the disease control rate (DCR). Both ORR and DCR are partly determined from a dichotomisation of the underlying continuous shrinkage in the total diameter of pre-specified target lesions (henceforth referred to as tumour size). To be classed as a success using ORR requires that tumour size shrinks by > 30%, with success using DCR requiring an increase of < 20% or a shrinkage. Generally using a dichotomised continuous variable loses statistical efficiency 4, and so the idea of directly using the tumour shrinkage itself as an endpoint has been proposed 5–7. However, RECIST also classifies patients as PD (and hence treatment failures in both ORR and DCR) if new tumour lesions are observed or if non-target lesions noticeably increase in size. Both of these possible events are associated with a poorer long-term survival prognosis, and using only the tumour shrinkage as the endpoint does not take into account patients who are treatment failures for these important reasons.

In addition, other possible outcomes may be of interest, such as toxicity. Because cytotoxic cancer treatments are toxic, patients in cancer trials often experience toxicities. At phase II, a new treatment would not be considered for a phase III trial if it caused substantial risk of death or toxicity, even if it caused tumour shrinkage. Bryant and Day 8 argue that toxicity should be considered in phase II cancer trials and extend the design of Simon 1 to include toxicities. Toxicities are generally graded from 1 to 4 using the Common Terminology Criterion for adverse events (http://ctep.cancer.gov/protocolDevelopment/electronic_applications/ctc.htm), with grades 3 and 4 being considered serious and often resulting in treatment being discontinued. We henceforth refer to grades 3 and 4 toxicities as ‘toxicity’. To complicate matters, once a patient experiences progressive disease or suffers a toxicity, they are usually removed from the trial, and their tumour shrinkage no longer measured.

To improve precision in estimation of a treatment's ORR or DCR, we consider a composite ‘success’ endpoint determined by (1) the change in tumour size; (2) the appearance of new lesions or increase in non-target lesion size; and possibly also (3) toxicity and/or death. This success endpoint therefore has both continuous and binary components. To be classified as a treatment success, a patient must be a success for the binary component (i.e. not have new tumour lesions), and their continuous component (tumour shrinkage) must be greater than a pre-defined threshold, which will depend on whether the treatment is cytotoxic or cytostatic. The probability of treatment success is equivalent to the ORR if toxicity or death are not considered and the threshold is 30%; similarly, it is equivalent to the DCR if the threshold is − 20%.

In trials comparing two treatments, and those comparing one or two treatments to historical data, it is of interest to estimate the various probability of treatment successes and to provide some measure of uncertainty for this estimate, for example, a confidence interval (CI). In this paper, we propose a method that we call the augmented binary approach. This uses the actual value of the observed tumour shrinkage (henceforth referred to as continuous tumour shrinkage), rather than just whether it is above a threshold, in order to reduce the uncertainty in the estimate of success probability. Consequently, the width of the CI for the probability of success can be reduced. This also increases power to detect differences between arms or to test a hypothesis comparing the treatment to historical data. The idea of testing hypotheses about binary outcomes using continuous data was originally suggested by Suissa 9. There, the binary endpoint was formed purely by a dichotomisation of a continuous variable, and each individual had an observed value for the continuous variable. The augmented binary method is a generalisation of Suissa's approach to a composite binary endpoint where complete tumour shrinkage data are not available for patients who are treatment failures for reasons (2) or (3) in the previous paragraph. The method leads to valid inference when the probability of dropout depends only on observed information (i.e. when data are missing at random (MAR)). Although trials are often not powered for a comparison of treatment success probabilities in the two arms, such a comparison is often made in randomised trials, and so we also consider the power of the augmented binary approach when this is carried out. We compare this power with those of a logistic regression approach and an approach proposed by Karrison *et al.* 6, which directly tests the continuous shrinkage using a nonparametric test.

## 2. Methods

### 2.1. Estimating success probability using the augmented binary method

We assume that the aim is to estimate the probability of success of a treatment, that is, the proportion of patients that have tumour shrinkage above some critical value (assumed for now to be 30 *%*) and do not fail for other reasons (i.e. new lesions, non-target lesions increase in size, toxicity or death).

During the trial, *n* patients are allocated to the treatment under consideration. Each patient has their sum of target lesion diameters measured at baseline. This quantity is measured halfway through the treatment and at the end of treatment for patients who remain in the study at these times. We denote these measurements for patient *i* as *z*_0*i*_ (baseline), *z*_1*i*_ (interim) and *z*_2*i*_ (end). We define non-shrinkage failure indicators: *D*_1*i*_ = 1 if patient *i* fails for a reason other than tumour shrinkage before the interim measurement, and *D*_2*i*_ = 1 if such a failure occurs between the interim measurement and the end of treatment. Henceforth, such failures are referred to as non-shrinkage failures. To allow the distribution of the continuous measurements to be approximated as a multivariate normal distribution, we use the log tumour-size ratio 5: 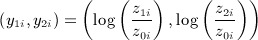. Note that complete responses, that is, a complete disappearance in tumour lesions, will have an undefined log tumour-size ratio. Instead, the lowest tumour-size ratio of all other patients can be substituted. If the proportion of complete responses is low, as is the case in most applications using RECIST, then the resulting deviation from the normality assumption does not affect the operating characteristics of methods assuming normality; if the proportion of complete responses is higher, then a more sophisticated model, such as one based on the censored normal distribution, could be used instead 10.

We define *S*_*i*_ as the observed composite success indicator for patient *i*. The value of *S*_*i*_ is 1 if *D*_1*i*_ = 0, *D*_2*i*_ = 0 and *y*_2*i*_ < log(0.7). In words, *S*_*i*_ is equal to 1 if patient *i* has a tumour shrinkage of more than 30 *%* at the end of treatment and no non-shrinkage failure. The value of *S*_*i*_ is missing if the patient drops out of the trial for a reason other than one of the failure criteria.

For the augmented binary approach, models must be specified for the tumour shrinkage and the probability of non-shrinkage failure. The tumour shrinkage is modelled using a bivariate normal model: 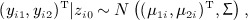
1

where *μ*_1*i*_ = *α* + *γz*_*i*0_, *μ*_2*i*_ = *β* + *γz*_*i*0_. Tumour size measurements that are missing because of non-shrinkage failures are treated as MAR. This is a valid assumption if the probability of non-shrinkage failure depends only on the previously observed tumour size. Additional covariates can be included in the tumour-shrinkage model to make the MAR assumption more plausible. Model eq (1) assumes that mean logarithm of the shrinkage is determined by baseline tumour size and the time of the observation (i.e. interim or end). An unstructured covariance matrix, Σ, is used. This class of model can be fitted in R 11 using the gls function in the nlme library 12.

For the non-shrinkage failure process, we separately model the probability of non-shrinkage failure before the interim (i.e. the probability of *D*_*i*1_ = 1) and the conditional probability of non-shrinkage failure between interim and the end given that the patient survived to the interim (i.e. the probability of (*D*_*i*2_ = 1 | *D*_*i*1_ = 0)). Logistic regression is used for both models: 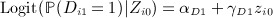
2

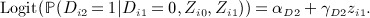
3

Let *θ* be the vector of parameters from the tumour shrinkage model,, and the non-shrinkage failure models, and. Using the aforementioned parameterisations, we have that *θ* is of length 10 (three parameters for *μ*_1_ and *μ*_2_, three for the covariance matrix Σ and two each in the two non-shrinkage failure models). The probability of success for patient *i*, with baseline tumour size *z*_0*i*_, is as follows: 
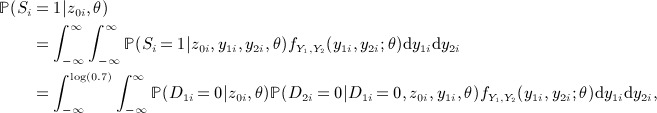
4

where 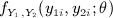 is the pdf of the bivariate normal distribution from Equation 1.

The mean success probability of the treatment is 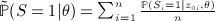, which can be estimated by 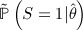, where 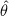 is the maximum likelihood estimator of *θ*. To get a CI for this probability, we transform to the log-odds scale, 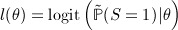. A CI for *l*(*θ*) requires an estimate of the variance of 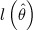, which we obtain using the delta method: 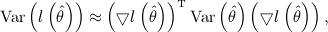
5

where 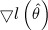 is the vector of partial derivatives of *l*(*θ*) with respect to *θ* evaluated at 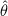. These partial derivatives can be approximated using the finite difference method. The parameters of the tumour size model and two non-shrinkage failure models are distinct, and so Var 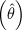, the covariance matrix of 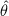, is block diagonal, that is, the covariance between parameter estimates from the different models is zero.

An approximately (1 − *α*)100*%* CI for 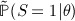 is 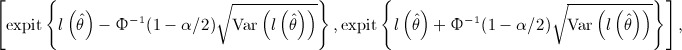


where Φ^ − 1^(1 − *α* / 2) is the 100(1 − *α* / 2) percentile of the standard normal distribution and expit is the inverse logit function.

For comparison, we also use the method of estimating the probability of success and a CI by using just the binary success indicators (i.e. the *S*_*i*_*s*). The CI is found using a Wilson score interval 13. This method is referred to as the ‘binary’ method.

### 2.2. Testing for a difference in success probability between two treatments

We assume that the trial proceeds as follows: 2*n* patients are recruited, with *n* randomised to each treatment. All patients are measured as in Section 2.1, and all definitions remain the same. The only difference is that a parameter representing the effect of treatment is included in models . Thus, the tumour shrinkage is modelled as follows: 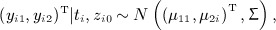
6

where *i* indexes the *i*th patient, *μ*_1*i*_ = *μ* + *β*_1_*t*_*i*_ + *γz*_*i*0_, *μ*_2*i*_ = *μ* + *δ* + *β*_2_*t*_*i*_ + *γz*_*i*0_, and *t*_*i*_ is the treatment indicator for patient *i*.

The models for non-shrinkage failure are as follows: 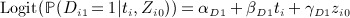
7

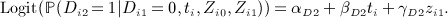
8

normal model:Let *θ* be the vector of parameters from the tumour shrinkage model,, and the non-shrinkage failure models, and. Using the aforementioned parameterisations, we have that *θ* is of length 14 (five parameters for *μ*_1_ and *μ*_2_, three for the covariance matrix Σ and three each in the two non-shrinkage failure models). Using the three models, the probability of success for a patient, 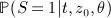, is as in Equation 4. We define the true mean difference in success probability, *m*(*θ*), as follows: 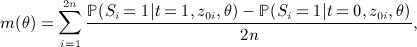
9

Formulating the difference in this way adjusts the analysis for a chance imbalance in baseline tumour size (or other covariates that may affect probability of success, if they are included in any of the models).

The estimated mean difference is 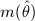. We use the Wald test for the null hypothesis that the true mean difference in success probabilities is zero. The variance of 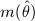 is again estimated using the delta method.

R code to apply the augmented binary method is given in the Supporting information.

For comparison, we consider a logistic regression that is fitted directly to the observed *S* ′ *s*, with a parameter for the baseline tumour size and a parameter for the treatment effect. A Wald test of the treatment effect is used as the test statistic for the difference in success probability between arms. Patients in which success status is missing are not included in the analysis. This method is subsequently referred to as the ‘logistic-regression’ method.

Also considered is the method of Karrison *et al.* 6. The log tumour shrinkage of patients, *y*_*i*2_, is directly compared between arms using a nonparametric test. For patients who suffer a non-shrinkage failure, their log tumour shrinkage is set to the worst observed value from all other patients. Patients who drop out for non-shrinkage reasons are not included in the analysis.

## 3.Simulation study

To compare the operating characteristics of the augmented binary approach with those of estimating the success probability using just the binary success data (for non-comparative trials) and the logistic regression approach and Karrison's method (for comparative trials), we conducted a simulation study. We describe the simulation setup first for non-comparative trials and then for comparative trials. In all simulations, *n* = 50 or *n* = 75 patients are randomised to each treatment. This represents sample sizes seen in recent randomised phase II cancer trials.

### 3.1. Simulation setup for non-comparative trials

We assume each patient's baseline tumour size is uniformly distributed between 5 and 10 cm. We denote the mean log tumour size ratio at the final endpoint as *δ*_1_. We generate data assuming that for a given treatment, with treatment effect *δ*_1_, the distribution of the log tumour size ratio at the interim and final endpoint is multivariate normal with mean (0.5*δ*_1_,*δ*_1_) and covariance matrix 
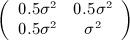
.

The models used to determine the probabilities of non-shrinkage failure before and after interim for the simulated data are those given by Equations 2 and 3. For all simulation scenarios, the values of *γ*_*D*1_ and *γ*_*D*2_ are set to the same value, *γ*_*D*_; similarly, *α*_*D*1_ = *α*_*D*2_ = *α*_*D*_.

A similar model is independently used to simulate dropout due to non-failure reasons (subsequently referred to as dropout). We denote the intercept and effect of tumour size in this model as *α*_*O*_ and *γ*_*O*_, respectively. This model determines if an individual's non-shrinkage failure status and continuous tumour shrinkage are missing at future observation times. Thus, if in a particular interval an individual is simulated to suffer a non-shrinkage failure and also to drop out, they are recorded as having dropped out.

In the simulation study, the values of *δ*_1_, *σ*^2^, *α*_*D*_, *γ*_*D*_, *α*_*O*_ and *γ*_*O*_ are varied. The performance of the augmented binary method is assessed in terms of bias and coverage from 5000 simulation replicates. Also, we estimate the reduction in the width of the 95*%* CI compared with the binary methods.

### 3.2. Simulation setup for comparative trials

For comparative trials, we denote the mean log tumour size ratio at the final endpoint as *δ*_0_ and *δ*_1_ in the control and experimental arms, respectively. The values for *δ*_0_ and *δ*_1_ are determined by two parameters, *x* and *ψ*: 
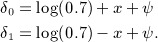


The value of 2*x* determines the difference in the mean log tumour size ratio of the two treatments, and the value of *ψ* reflects the effectiveness of the control treatment. When *ψ* = 0, the two mean shrinkages are symmetric around log(0.7), corresponding to a 30 *%* shrinkage, which is the dichotomisation point used in the ORR endpoint.

The data were then simulated as in the previous section. The models for simulating non-shrinkage failures and dropout also include treatment effect parameters *β*_*D*_ and *β*_*O*_.

The augmented binary approach was compared with fitting a logistic regression model to the binary success data and also with Karrison's method applied using the Wilcoxon rank-sum test. For each simulation study, 5000 datasets were simulated for each parameter combination. For a true type I error rate of 0.05, this gives a Monte Carlo standard error for the estimated type I error rate of 0.003.

### 3.3. Operating characteristics of augmented binary approach for non-comparative trials

Table 1 summarises the operating characteristics of the augmented binary approach for different parameter values. In most situations, the augmented binary method considerably reduces the width of the CI compared with the binary method. For example, with *n* = 75 and the baseline simulation scenario, the augmented binary method reduces the average width of the 95 *%* CI by 17 *%*. To obtain a similar average width using just the binary data, a sample size of 1.17^2^ = 1.37 times bigger, that is, around 103, would be needed. This figure of 37 *%* is very similar to the loss in information from dichotomising a continuous treatment outcome (e.g. in Wason *et al.* 7).

**Table I:** Operating characteristics of augmented binary method (Aug Bin) in comparison with just using the binary success data (Bin).

Scenario	*n*	true 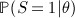	Mean 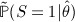		Estimated coverage		Reduction in 95 *%*
			Bin	Aug Bin	Bin	Aug Bin	CI width
Baseline	50	0.334	0.336	0.334	0.948	0.944	16.5 *%*
Baseline	75	0.334	0.336	0.334	0.950	0.948	17.3 *%*
*δ*_1_ = 0	50	0.241	0.242	0.240	0.933	0.941	22.1 *%*
*δ*_1_ = 0	75	0.241	0.241	0.240	0.958	0.943	22.5 *%*
*δ*_1_ = 0.18	50	0.197	0.196	0.195	0.951	0.931	26.6 *%*
*δ*_1_ = 0.18	75	0.197	0.197	0.197	0.944	0.924	27.3 *%*
*σ* = 2	50	0.333	0.332	0.335	0.945	0.941	17.1 *%*
*σ* = 2	75	0.333	0.333	0.335	0.947	0.949	17.9 *%*
(*μ*_*D*_,*γ*_*D*_) = ( − 2.5,0.2)	50	0.293	0.293	0.295	0.942	0.950	13.6 *%*
(*μ*_*D*_,*γ*_*D*_) = ( − 2.5,0.2)	75	0.293	0.292	0.293	0.940	0.945	14.1 *%*
(*μ*_*O*_,*γ*_*O*_) = ( − 2.15,0)	75	0.334	0.326	0.332	0.953	0.948	17.5 *%*
(*μ*_*O*_,*γ*_*O*_) = ( − 2.9,0.1)	75	0.334	0.333	0.333	0.949	0.952	17.4 *%*

All estimates based on 5000 replicates. Simulation parameters are described in Section 3.1. Baseline scenario corresponds to *δ*_1_ = − 0.356,*σ* = 1,*μ*_*D*_ = − 1.5,*γ*_*D*_ = 0,*μ*_*O*_ = − ∞ ,*γ*_*O*_ = 0 (i.e. no dropout); non-baseline scenarios are as in the baseline scenario except for the specified difference.

Although there is a clear reduction in CI width, the augmented binary method appears in two scenarios to have slightly below nominal coverage. These two scenarios are also the scenarios where the power gain is greatest. The worst coverage observed (92.4 *%*) is when *δ*_1_ = 0.18, which corresponds to a median increase in tumour size between baseline and final time points of 20 *%*. We changed the dichotomisation threshold to 0*%*, which improved coverage to 94.5 *%*. In practice, before the trial started, if one expected a treatment to result in a low average tumour shrinkage, or an average increase in tumour size, a more suitable dichotomisation threshold should be used, such as that of the DCR endpoint.

Interestingly, in scenarios when there is dropout (the last two rows in Table 1), the augmented binary approach gives roughly the same average reduction in 95*%* CI width as occurs in the analogous scenario with no dropout. This is despite a decrease in the number of patients with complete data. This suggests that the fact that the augmented binary approach allows inclusion of interim data for patients who dropout between the interim and the end improves the precision of the estimated probability of success. This is only the case if there is some correlation between the interim measurement and the final measurement.

### 3.4. Comparison of approaches for testing the treatment effect in comparative trials

#### 3.4.1. Comparison at mean log tumour ratio varies

We first investigate the case where the non-shrinkage failure process does not depend on treatment or tumour size, that is, *β*_*D*_ = 0 and *γ*_*D*_ = 0. The value of *α*_*D*_ is set to − 1.39, corresponding to a 20 *%* chance of failing between baseline and interim, and 20 *%* between interim and the final observation. We assume no dropout. Further, we set *ψ* to 0, corresponding to the mean log tumour size ratio at the final timepoint of the two treatments being symmetric around log(0.7). We varied *x* in increments of 0.05 between 0 and 0.40.

**Figure 1:**
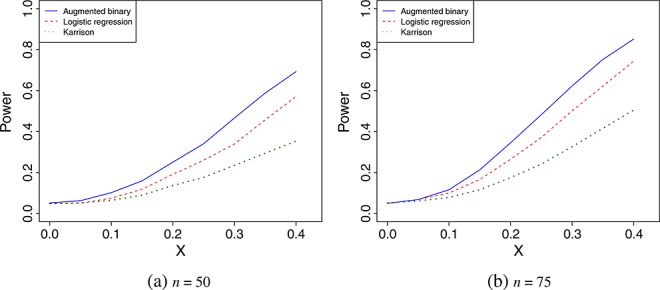
Power of the three methods for *n* = 50 and *n* = 75 as the difference in mean log tumour size ratio, measured by *x*, varies.

Figure 1 shows the power of the three methods as *x* varies. It shows there is a consistent power advantage by using the augmented binary approach. The worst power is shown by Karrison's method. The type I error rate of all three methods (i.e. the power when *x* = 0) is controlled at the nominal level of 0.05.

**Figure 2:**
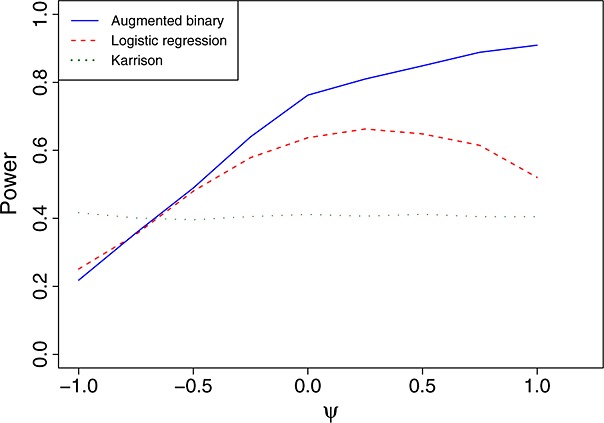
Power of the three methods for *n* = 75 and *x* = 0.35 as *ψ* varies.

Figure 2 shows the power of the three methods for *n* = 75 and *x* = 0.35 as the value of *ψ* changes. The mean shrinkage of both treatments decrease as *ψ* increases. Because the Wilcoxon rank-sum test statistic uses only ranks, the value of *ψ* does not affect the power of Karrison's method. For values of *ψ* < − 0.65, the augmented binary approach has the worst power of the three methods. As *ψ* increases, the power of the augmented approach increases, whereas the power of logistic regression reaches a peak at *ψ* = 0.25 and then decreases.

Table 1 in the Supporting information summarises the type I error rates as the value of *ψ* changes. For the augmented binary and logistic regression methods, they are generally slightly inflated for negative *ψ* and deflated for positive *ψ*. The deviation from 0.05 is generally greater for the logistic regression than for the augmented binary approach (the type I error rate of the former when *ψ* = 1 is 0.026 when *n* = 50 and 0.038 when *n* = 75). This is consistent with previous research showing that when there are fewer than five ‘events’ (in this case, failures) per parameter in a logistic regression, the standard error can be poorly estimated 14. Karrison's method controls the type I error rate at the nominal level for all values of *ψ*.

The aforementioned results show that the augmented binary approach has low power for large negative values of *ψ*. A negative value of *ψ* means that both treatments are, on average, effective at shrinking tumours, and so most patients surviving will have a tumour shrinkage far above the threshold for success. If this is the case, then using the exact tumour shrinkage will not improve estimation of the probability of success as much as it will when the mean shrinkage is close to the threshold. However, this only explains why the power of the augmented binary approach should be equal to that of the logistic regression approach, although it appears to be slightly lower in Figure 2. This slightly lower power could be due to the augmented binary approach requiring estimation of a greater number of parameters (14 for the augmented binary method versus 3 for the logistic regression method).

When both treatments are highly effective, a better dichotomisation threshold would be one that creates a more equal balance between the number of successes and failures. We investigated the power of the augmented binary and logistic regression methods for (*x*,*ψ*) = (0.35, − 1) as the dichotomisation threshold was varied (Karrison's method was not considered because its power does not depend on the dichotomisation threshold). These parameters correspond to a median shrinkage of around 63 *%* using the control treatment and around 82 *%* using the new treatment. The results are shown in Figure 1 of the Supporting information. They show that the power of both approaches increases as the dichotomisation threshold decreases (i.e. a greater tumour shrinkage is required to declare success) and the augmented binary approach becomes more powerful than the logistic regression method. This indicates that if large average tumour shrinkages are expected, the dichotomisation threshold should be lowered – not only will this cause both methods to gain power but also will make the augmented binary method more powerful than the logistic regression method.

#### 3.4.2. Comparison as probability of non-shrinkage failure varies

**Figure 3:**
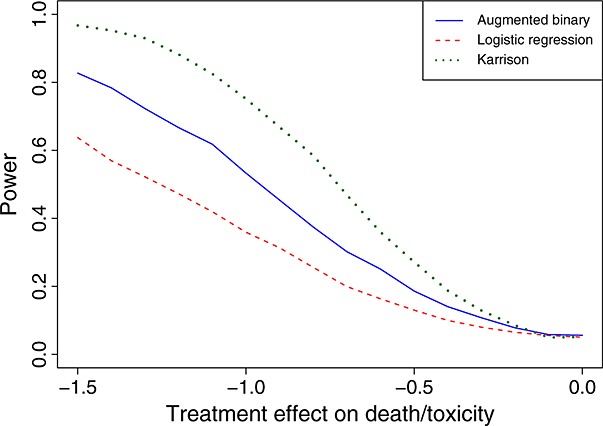
Power of the three methods for *n* = 75 and (*x*,*ψ*) = (0,0) (i.e. *δ*_0_ = *δ*_1_ = log(0.7)) as *β*_*D*_, the effect of treatment on non-shrinkage failure, changes.

We next investigated the relative power of the three methods as the parameters used to generate the non-shrinkage failure process were varied. Both treatments were assumed to have a median tumour shrinkage of 30*%*, that is, (*x*,*ψ*) = (0,0). Figure 3 shows the power as *β*_*D*_, the effect of treatment on the log-odds of non-shrinkage failure, changes. Negative values of *β*_*D*_ mean that the probability of non-shrinkage failure is lower when using the new treatment compared with the control treatment. We set *γ*_*D*_, the effect of the tumour size on probability of non-shrinkage failure, to be zero. The results show that Karrison's method is the most powerful in this situation, with the augmented binary approach in second place. Karrison's approach of setting all patients who die or suffer toxicity to the worst possible outcome makes the approach very powerful when there is a difference in probability of non-shrinkage failure between the two arms. The augmented binary approach has noticeably higher power than logistic regression. This is likely to be because the augmented binary approach models the probability of non-shrinkage failure before the interim and after the interim separately, whereas the logistic regression method only models whether or not a non-shrinkage failure occurred and does not distinguish between events before and after the interim.

Figure 2 in the Supporting information shows the power for *β*_*D*_ = − 1 as *γ*_*D*_ varies. The power of all three approaches appears to be insensitive to *γ*_*D*_ (although there is a slight decrease as *γ*_*D*_ increases). Karrison's method consistently shows the highest power, followed by the augmented binary approach.

We also investigated a scenario where the new treatment has a higher mean tumour shrinkage and also a lower probability of non-shrinkage failure (*x* = 0.175, *β*_*D*_ = − 0.5, *γ*_*D*_ = 0 and *α*_*D*_ = − 1.155). In this scenario, the augmented binary approach has a slightly higher power than Karrison's method (0.688 compared with 0.642). More generally, the most powerful method will depend on the relative magnitudes of the effects of the new treatment on mean tumour shrinkage and on probability of non-shrinkage failure.

### 3.5. Sensitivity analyses

We wished to assess the sensitivity of the operating characteristics to two assumptions made by the augmented binary method. The first assumption is that the probability of non-shrinkage failure depends only on the previous tumour size observation; the second is that the various reasons for non-shrinkage failure can be included together in one binary category; and the third is that of normality of the log tumour size ratio. A full description of the methods, together with simulation results, is given in the Supporting information. Generally, the augmented binary method was robust to all three assumptions.

## 4.Case study

To illustrate the use of the augmented binary approach, we applied it to data from the CAPP-IT trial (discussed by Corrie *et al.* 15). CAPP-IT was a multi-centre, randomized, placebo-controlled study assessing the effect of pyridoxine on reducing dose medications when treating cancer patients with capecitabine. Hand-foot syndrome is a common adverse effect of capecitabine, and its occurrence often results in treatment being modified (i.e. delayed or discontinued). In the trial, 106 patients who had been assigned to palliative single-agent capecitabine chemotherapy were randomized to receive pyridoxine or placebo (53 in each arm). The primary outcome was the probability of capecitabine dose modification, with tumour response a secondary outcome. The trial was not powered to detected differences between tumour response in the two arms, so we consider the two arms separately. Patients were assessed every 12 weeks until disease progression, toxicity (including hand-foot syndrome) or dropout for other reasons. We analyse the data as if the endpoint of interest were the probability of success at 24 weeks. We thus have a maximum of three tumour size measurements per patient: at baseline, halfway through treatment and at the end of treatment.

As in the simulation study, we define a patient as successful if no toxicity or death occurs, no new lesions develop and the tumour size shrinkage between baseline and the final observation is greater than 30*%*. Because patients were recorded as treatment failures if their tumour size increased by 20 *%* or more between the baseline and interim measurements, we also include this as a failure criterion. With this addition, the probability of success is similar to Equation 4, except that success requires a first stage log tumour shrinkage ratio of less than 1.2: 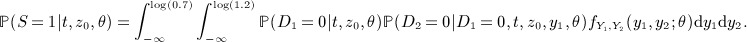


Table 2 shows the numbers of patients, successes and patients with unknown success status.

**Table II:** Summary of number of successes and failures on each arm.

	Placebo	Pyridoxine
Treatment successes	6	5
Failures due to less than required tumour shrinkage	14	23
Failures due to non-shrinkage reasons	25	21
Number with unknown success status due to dropout	8	9
Total patients *	49	50

Only patients who did not drop out of the trial before the baseline tumour measurement are included.

* Note that categories are not mutually exclusive, that is, patients can fail for both tumour shrinkage and non-tumour shrinkage reasons.

We estimated the probability of success, together with a 95 *%* CI, for each arm separately. The non-shrinkage failure models are as in Equations 2 and 3. The model for tumour shrinkage is the same as in Equation 1. The augmented binary method is compared with estimating the probability of success from the binary data alone. All patients with baseline tumour size data were included in the augmented binary analysis, whereas only complete cases could be considered using the binary method.

Table 3 shows the estimated probability of success and 95 *%* CI for both arms using the augmented binary and binary methods. We consider three possible dichotomisation thresholds: 0.7, corresponding to a 30 *%* shrinkage in tumour size required for success; 1, corresponding to any shrinkage required for success; and 1.2, corresponding to a shrinkage or increase of less than 20 *%* required for success. The first and third of these are the thresholds used in the objective response rate and the disease control rate, respectively.

**Table III:** Estimated probability of success and 95 *%* CIs from binary and augmented binary methods for the two treatments in the case study.

		Estimated 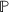 (success)		95 *%* CI	
Dichotomisation threshold	Treatment	Binary	Augmented binary	Binary	Augmented binary
0.7	Placebo	0.146	0.143	(0.069–0.284)	(0.080–0.241)
0.7	Pyridoxine	0.122	0.068	(0.053–0.255)	(0.034–0.134)
1	Placebo	0.171	0.239	(0.085–0.313)	(0.149–0.360)
1	Pyridoxine	0.171	0.191	(0.085–0.313)	(0.115–0.299)
1.2	Placebo	0.220	0.285	(0.120–0.367)	(0.184–0.413)
1.2	Pyridoxine	0.220	0.262	(0.120–0.367)	(0.170–0.380)

Table 3 shows that the augmented binary method can change the estimate of the success probability considerably in some cases. The largest change is a reduction in the estimated success probability from 0.122 to 0.068 for pyridoxine with a dichotomisation threshold of 0.7. In this case, three successful patients had tumour shrinkages very close to the dichotomisation threshold, whereas just one treatment failure was close to being a success. In other cases, the two methods give similar estimates. In all cases, the 95 *%* CIs from the two methods overlap. The augmented binary method gives reductions in the width of the CI in all cases. In the case where the estimates are most similar (placebo with a dichotomisation threshold of 0.7), the augmented binary method gives a 25 *%* reduction in the width of the CI – a considerable reduction.

## 5.Discussion

In this paper, we have proposed and assessed the augmented binary method, which makes inference about a composite success outcome defined by a continuous outcome and a binary outcome, using the continuous component to improve precision. This method is motivated by phase II cancer trials, where tumour response is a composite endpoint defined by continuous tumour shrinkage and binary non-shrinkage failure indicators, such as whether new lesions are observed. We find that in general, the augmented binary approach improves inference about the probability of success considerably over methods that only consider whether the continuous tumour shrinkage is above a threshold.

There are several issues for consideration before using the augmented binary method. One issue is whether the more complicated methodology is worth applying for the gains seen. We show that the information gain from using the augmented binary method is comparable with the gain seen from modelling a continuous outcome directly rather than dichotomising it. There is a strong consensus amongst statisticians that it is a bad idea to dichotomise continuous outcomes. However, generally this consensus is seen in situations where alternative continuous models are easy to apply, such as use of a linear model instead of a logistic regression. We have included code in the Supporting information, which we hope will reduce the difficulty of implementing the augmented binary method. A second issue is how the sample size for a trial using the augmented binary method should be chosen. Because several endpoints are of interest at phase II, we believe the sample size should be chosen as if the trial were to be analysed using traditional methods. Then, using the augmented binary approach provides extra precision on the estimated success probability or higher power for a comparison between two arms. Because of the number of parameters used, we would suggest that the method only be used for reasonably large sample sizes, at least 50 per arm. A third issue is that in certain situations, the augmented binary approach does not add any power – for instance, when the probability of success is very high. Because of this, the dichotomisation threshold for the tumour shrinkage is very important. A suitable dichotomisation threshold should be pre-specified so that the expected probability of success is not too low or high. For example, if few partial responses are expected, the disease control rate would be a more suitable choice of endpoint than the response rate. In these situations, the simulations show clear gains from use of our method.

For phase II trials with fewer than 50 patients, the number of parameters in the model could be reduced by making additional assumptions, for example, that the effect of the tumour size on the probability of non-shrinkage failure is the same in models  and . Alternatively, *p*-values and confidence intervals could be calculated by using a bootstrap procedure.

A recently published paper 16 proposes an alternative method for using continuous tumour information in randomized comparative phase II trials. Unlike in our paper and in other trials using RECIST as an outcome, the outcome of interest is overall survival. Historical data are used to estimate the effect of tumour size change on overall survival in the absence of treatment. It is assumed that the association between tumour shrinkage and overall survival in untreated patients is the same in the historical and current datasets and that any treatment effect on overall survival is captured by the effect of treatment on change in tumour size. These assumptions enable the difference in expected overall survival in the treated and untreated group implied by the observed difference in tumour shrinkage to be derived. Finally, the test statistic for the treatment effect on overall survival is the sum of two test statistics: one based on this difference in expected overall survival and one based on the observed difference in overall survival. The paper shows that the approach is promising, with required sample sizes considerably smaller than those for trials using binary tumour response. Note that the method neither explicitly take into account treatment failures for non-shrinkage reasons, such as new lesions appearing, nor allow for interim tumour-size measurements to be made, although extensions to allow these may be possible.

One assumption made by the augmented binary approach is that the probability of non-shrinkage failure depends only on the most recently observed value of the tumour size. This is a strong assumption, because the tumour may change in size considerably between observations and thus cause the probability of non-shrinkage failure to change over time. We investigated, using simulations, the effects of deviating from this assumption (Supporting information) and found no evidence that the method was sensitive to this assumption. If there is, nevertheless, still a concern about the effect about a possible violation of this assumption, an alternative to the model we have proposed would be a shared parameter model 17. In this latter model, the tumour size process and non-shrinkage failure process depend on common unobserved random effects. This enables the hazard of failure to depend on the current underlying tumour size. In the same way, dropout for other reasons could also be allowed to depend on current underlying tumour size.

In many phase II cancer trials, patients are followed up until they progress, with tumour measurements taken at regular intervals. Tumour response is then analysed by considering the best observed response seen before progression. In this paper, we focus on response at a fixed timepoint (for example, when treatment ends). We believe this is a better choice than the best observed response for several reasons: (1) the response at a fixed timepoint has previously been shown to be more informative for overall survival than the best observed response 18; (2) there is a high measurement error in assessing tumour size, and the best observed response is likely to be more susceptible to measurement error; (3) the response at a fixed timepoint will often take considerably less time to observe than the best observed response, so trials can be conducted more quickly. If patients are followed up to progression, then instead of analysing the best observed response, a more natural analysis would be to fit a model to the time-to-progression data and to assess tumour response at a fixed timepoint as a secondary analysis. If it is of interest to assess the best observed response, it would be possible to extend our methodology to do this. A similar model, with more timepoints, would be fitted to the continuous tumour data. It would be necessary to make simplifying assumptions to reduce the number of parameters in this model, for example, by imposing additional structure on the covariance matrix. For the non-shrinkage failure data, a time-to-event model such as a Cox model could be fitted. One would then simulate the best observed response by simulating patient data from the two models. A CI for this estimate could be found using a method such as bootstrapping. This would be extremely computationally intensive, and alternative quicker approaches are a topic of further research.

## References

[1] Simon R (1989). Optimal two-stage designs for phase II clinical trials. Controlled Clinical Trials.

[2] Rubinstein L, Crowley J, Ivy P, LeBlanc M, Sargent D (2009). Randomized phase II designs. Clinical Cancer Research.

[3] Eisenhauer EA, Therasse P, Bogaerts J, Schwartz LH, Sargent D, Ford R, Dancey J, Arbuck S, Gwyther S, Mooney M, Rubinstein L, Shankar L, Dodd L, Kaplan R, Lacombe D, Verweij J (2009). New response evaluation criteria in solid tumours: revised RECIST guideline (version 1.1). European Journal of Cancer.

[4] Altman D, Royston P (2006). The cost of dichotomising continuous variables. BMJ.

[5] Lavin P (1981). An alternative model for the evaluation of antitumor activity. Cancer Clinical Trials.

[6] Karrison T, Maitland M, Stadler W, Ratain M (2007). Design of phase II cancer trials using a continuous endpoint of change in tumour size: application to a study of sorafenib and erlotinib in non-small-cell lung cancer. JNCI.

[7] Wason J, Mander A, Eisen T (2011). Reducing sample sizes in two-stage phase II cancer trials by using continuous tumour shrinkage endpoints. European Journal of Cancer.

[8] Bryant J, Day R (1995). Incorporating toxicity considerations into the design of two-stage phase II clinical trials. Biometrics.

[9] Suissa S (1991). Binary methods for continuous outcomes: a parametric alternative. Journal of Clinical Epidemiology.

[10] Wason J, Mander A (2012). The choice of test in phase II cancer trials assessing continuous tumour shrinkage when complete responses are expected. Statistical Methods in Medical Research.

[11] R Development Core Team (2011). R: A language and environment for statistical computing. R Foundation for Statistical Computing.

[12] Pinheiro J, Bates D, DebRoy S, Sarkar D, R Development Core Team (2011). nlme: Linear and nonlinear mixed effects models.

[13] Wilson E (1927). Probable inference, the law of succession, and statistical inference. Journal of the American Statistical Association.

[14] Peduzzi P, Concato J, Kemper E, Holford T, Feinstein A (1996). A simulation study of the number of events per variable in logistic regression analysis. Journal of Clinical Epidemiology.

[15] Corrie PG, Bulusu R, Wilson CB, Armstrong G, Bond S, Hardy R, Lao-Sirieix S, Parashar D, Ahmad A, Daniel F, Hill M, Wilson G, Blesing C, Moody AM, McAdam K, Osborne M A randomised study evaluating the use of pyridoxine to avoid capecitabine dose modifications. British Journal of Cancer.

[16] Jaki T, Andre V, Su T-L, Whitehead J (2013). Designing exploratory cancer trials using change in tumour size as primary endpoint. Statistics in Medicine.

[17] Henderson R, Diggle P, Dobson A Joint modelling of longitudinal measurements and event time data. Biostatistics.

[18] An MW, Mandrekar SJ, Branda ME, Hillman SL, Adjei AA, Pitot HC, Goldberg RM, Sargent DJ (2011). Comparison of continuous versus categorical tumor measurement-based metrics to predict overall survival in cancer treatment trials. Clinical Cancer Research.

