# Supplementary material for: Using continuous data on tumour measurements to improve inference in phase II cancer studies
Source: Stat Med. 2013 Jun 18;32(26):4639–50. doi: 10.1002/sim.5867 (PMC4282550; doi:10.1002/sim.5867)
Supplement: Supplementary file 1 [file sim0032-4639-SD1.pdf]

Supplementary material for "Using continuous data on tumour measurements to improve inference in phase II cancer studies" by James Wason and Shaun Seaman

1 Supplementary methods

1.1 Sensitivity to endpoint assumed

We wished to assess the sensitivity of each of the three methods to the model used to simulate the tumour shrinkage. Recall that (z_{i0}, z_{i1}, z_{i2}) represents the baseline, interim, and final values of the tumour size. The simulation results in section 3.4 of the main paper assume that the logarithm of the tumour size ratios, $\left(\log\left(\frac{z_{i1}}{z_{i0}}\right), \log\left(\frac{z_{i2}}{z_{i0}}\right)\right)$, are distributed as a bivariate normal random variable with constant covariance matrix.

We considered three alternatives to this:

1. The ratios of the tumour sizes, $\left(\frac{z_{i1}}{z_{i0}}, \frac{z_{i2}}{z_{i0}}\right)$ is distributed as a bivariate normal distribution with constant covariance matrix. In order to avoid situations where a ratio for an individual was below 0, we chose smaller values for the components of the covariance matrices. The covariance matrix used was $\begin{pmatrix} 0.125 & 0.125 \\ 0.125 & 0.25 \end{pmatrix}$. Under the null, the mean tumour ratio was set to 0.7, corresponding to a shrinkage of 30%. Under the alternative, the control treatment had a mean of $0.7 + 0.35 \times 0.25$, and the active treatment had a mean of $0.7 - 0.35 \times 0.25$.

2. The differences in the absolute tumour size $(z_{i1} - z_{i0}, z_{i2} - z_{i1})$ are assumed to be independent normal random variables with a standard deviation of 1. Under the null, the mean is chosen to give a mean tumour shrinkage between baseline and the final observation of 30%. Under the alternative, the means of the two treatments are symmetric around this mean.

3. The log tumour size ratio is a bivariate normal random variable, but the covariance matrix for an individual is $\begin{pmatrix} 0.5(\frac{z_{i0}}{\mathbb{E}(z_{i0})})^2 & 0.5(\frac{z_{i0}}{\mathbb{E}(z_{i0})})^2 \\ 0.5(\frac{z_{i0}}{\mathbb{E}(z_{i0})})^2 & (\frac{z_{i0}}{\mathbb{E}(z_{i0})})^2 \end{pmatrix}$. Thus, the standard deviation of the log tumour size ratio increases linearly with the baseline tumour size, but the average standard deviation across all patients is as before. The means of the two treatments are chosen as in the main paper.

2 Supplementary figures and tables

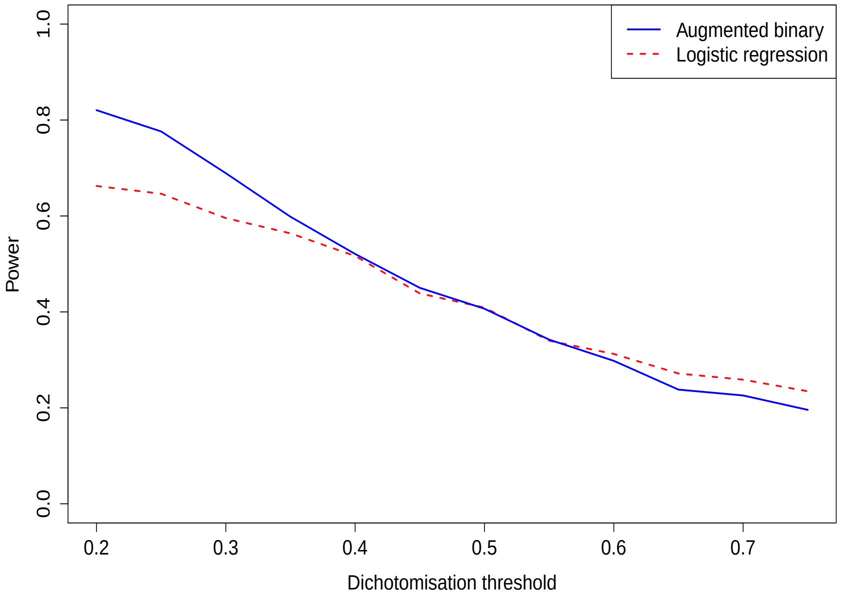

Figure 1: Power of the augmented binary and logistic regression methods for $n = 75$ for $\psi = -1$, $x = 0.35$ as the dichotomisation threshold changes. The traditionally used 30% threshold corresponds to 0.7 in the figure.

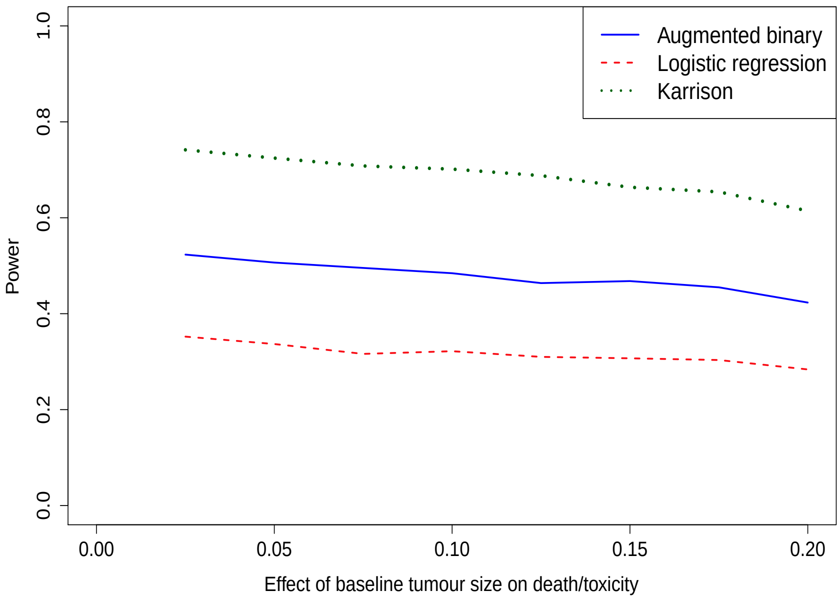

Figure 2: Power of the three methods for $n = 75$, $\delta_0 = \delta_1 = \log(0.7)$ and $\beta = 1$, as γ_D, the effect of tumour size on probability of failure, changes.

	ψ	Type-I error rate		
		Binary	Augmented binary	Karrison
	-1	0.055	0.052	0.048
	-0.75	0.063	0.061	0.052
	-0.5	0.063	0.060	0.046
n=50	-0.25	0.050	0.053	0.048
	0	0.046	0.054	0.048
	0.25	0.046	0.054	0.045
	0.5	0.043	0.048	0.055
	0.75	0.040	0.048	0.055
	1	0.026	0.050	0.050
	-1	0.049	0.052	0.047
	-0.75	0.047	0.058	0.052
	-0.5	0.055	0.061	0.050
n=75	-0.25	0.056	0.053	0.053
	0	0.048	0.048	0.052
	0.25	0.046	0.047	0.051
	0.5	0.049	0.046	0.048
	0.75	0.040	0.049	0.049
	1	0.038	0.053	0.049

Table 1: Estimated type-I error rate for $x = 0$ and varying values of ψ (section 3.4 in main paper).

3 Sensitivity analyses

The augmented binary approach makes several assumptions. We conducted a sensitivity analysis to three of these assumptions: 1) the hazard of non-shrinkage failure depends only on the most recent tumour size observation; 2) the various binary reasons for failure (new lesions, toxicity and death) can be combined and modelled as one process; and 3) the log tumour size ratios are bivariate normally distributed with constant covariance matrix.

3.1 Sensitivity to assumption that probability of failure depends on most recent tumour size observation

To investigate the sensitivity of the augmented binary method to assumption 1) we simulated tumour shrinkage data and failure data for patients as if they were observed at four post-baseline timepoints (referred to in the following text as timepoints 1, 2, 3, and 4) instead of two. Timepoints 2 and 4 are the interim and final timepoints from the two timepoint trial. The tumour size data was simulated assuming that the log tumour size ratios were multivariate normal with mean $(0.25\delta_1, 0.5\delta_1, 0.75\delta_1, \delta_1)$, and covariance matrix $\begin{pmatrix} 0.25 & 0.25 & 0.25 & 0.25 \\ 0.25 & 0.5 & 0.5 & 0.5 \\ 0.25 & 0.5 & 0.75 & 0.75 \\ 0.25 & 0.5 & 0.75 & 1 \end{pmatrix}$. The failure model was the same as used in the main paper, except extended to four time points.

We simulated non-comparative data with $n = 75$, $\delta_1 = \log(0.7)$, and no dropout for non-failure reasons. The parameters used in the failure model, α_D and γ_D were varied with $(\alpha_D, \gamma_D) = \{(-2, 0.1) \text{ or } (-2.75, 0.2)\}$. The positive γ_D parameters mean that patients with large tumour sizes at the previous timepoint are more likely to fail at the next timepoint than are those with smaller tumour sizes.

For each simulation replicate, the shrinkage data and failure data were simulated for all four timepoints. Then the data on timepoints 1 and 3 were thrown away, so that patients were only observed at baseline, interim and final timepoints. If a patient failed at timepoints 1 or 3 then they were recorded as failures at the interim and final timepoint respectively. The potential for bias here is that a patient's tumour size may be quite different at baseline and at timepoint 1 (say), but the augmented binary model will only use the baseline tumour size.

However, from 5000 simulation replicates, the coverage of the augmented binary approach was 94.46% when $\gamma_D = 0.1$ and 94.48% when $\gamma_D = 0.2$, which is not noticeably different from the results in the main paper. Thus it does not appear that the augmented

binary method is particularly sensitive to this assumption.

3.2 Sensitivity to modelling different reasons for failure as one category

To test the sensitivity of type-I error rate and power to assumption 2) we simulated data using separate models for the probability of new lesions and probability of death or toxicity. For the sensitivity analysis, we simulated data such that the parameters representing the effect of treatment on failure due to new lesions, and failure due to death/toxicity respectively have the same magnitude, but have opposite signs. This means that the overall probability of failure is the same in each treatment arm, but that the probability of new lesions is lower on one arm, and the probability of toxicity or death is lower on the other arm. The two differences cancel each other out, so that the probability of detecting a difference in success probability between treatments should be the type-I error rate. A range of treatment and tumour size parameter values were considered. For each set of parameter values, we estimated the probability of rejecting the null hypothesis when there was no difference in tumour shrinkage between treatments, and when the value of x was 0.35.

The results of this analysis are given in table 2. They show that violation of the assumption that two distinct reasons for non-shrinkage failure can be combined into one outcome has little noticeable effect on the type-I error rate of any of the three approaches. When the magnitude of the effect of tumour size is large, there is a small inflation in the type-I error rate of the logistic regression approach. In the extreme case where the $\gamma_D = 0.2$, and $\beta_D = 1$, the type-I error rate was estimated to be 0.067 for the logistic regression approach. The inflation in type-I error rate when using logistic regression is explained by the fact that the model fitted is mis-specified. The power of the augmented binary approach fell modestly when the direction of effects differed, whereas the power of the logistic regression approach fell more sharply. Thus, although the type-I error rate and power of the augmented binary approach are somewhat sensitive to the first assumption, the sensitivity is no greater than that of the logistic regression approach.

3.3 Sensitivity to normality of log tumour shrinkages

To test sensitivity to the assumptions made about the distribution of tumour shrinkages, we simulated tumour shrinkages using three different models: 1) the tumour-size ratios (rather than the logarithm of the ratios) are bivariate normal with constant covariance matrix; 2) the differences in tumour size between each visit are bivariate normal with constant covariance; 3) the log tumour-size ratios are bivariate normal, but the covariance

$\lvert\gamma_O\rvert$	$\lvert\beta_O\rvert$	Type-I error rate Logistic regression	Augmented binary	Karrison	Power for $x = 0.35$ Logistic regression	Augmented binary	Karrison
0	1	0.046	0.048	0.050	0.645	0.777	0.438
0.1	0	0.049	0.047	0.053	0.634	0.764	0.430
0.1	0.5	0.054	0.049	0.052	0.602	0.760	0.411
0.1	1	0.054	0.050	0.051	0.564	0.756	0.394
0.2	1	0.067	0.053	0.055	0.444	0.689	0.324

Table 2: Estimated type-I error rate and power when the treatment effect parameters in the models used to simulate failure due to new lesions and failure due to death/toxicity have the same magnitude, but different sign.

matrix is a linear function of the baseline tumour size. The estimated type-I error rate and power are shown in table 3.

The type-I error rate of the augmented binary approach shows no inflation for models 2) and 3). There was a small inflation (the observed type-I error rate was 0.057) for model 1). The power advantage of the augmented binary approach over logistic regression is sensitive to the endpoint used - using model 2) the power of the augmented binary approach (0.554) was lower than that of the logistic regression (0.580). These results indicate that when analysing real data, assessing the plausibility of the assumption of normality of the log tumour-size ratio is advisable. If there is evidence against the assumption of normality of the log tumour size ratio, the augmented binary approach can be easily modified so that a different function of the tumour shrinkage is used in the model represented in equation 1 in section 2.1 of the main paper.

Scenario	Type-I error rate Logistic regression	Augmented binary	Karrison	Power for $x = 0.35$ Logistic regression	Augmented binary	Karrison
1	0.052	0.057	0.053	0.786	0.828	0.571
2	0.041	0.049	0.045	0.580	0.554	0.374
3	0.049	0.048	0.049	0.678	0.769	0.440

Table 3: Estimated type-I error rate and power when the mechanism for generating tumour shrinkages differs. Scenario 1: the tumour-size ratios (rather than the logarithm of the ratios) are bivariate normal with constant covariance matrix; scenario 2: the differences in tumour size between each visit are bivariate normal with constant covariance; scenario 3: the log tumour-size ratios are bivariate normal, but the covariance matrix is a linear function of the baseline tumour size.

4 R code to fit augmented binary method

```
library(nlme)
library(R2Cuba)
library(boot)

integrand=function(logtumourratios,means_treated,means_untreated,Sigma,glm_dropout1,glm_dropout2,baselines)
{
#integrand evaluates the mean difference in success probability for a given log-tumour ratio.
#This function is called by probabilityofsuccess.

#get probabilities of not failing given patient is treated and given they are untreated:
n=length(baselines)
f1_treated=inv.logit(cbind(rep(1,n),baselines,rep(1,n))%*%glm_dropout1\$coefficients)
f2_treated=inv.logit(cbind(rep(1,n),exp(logtumourratios[1])*baselines,rep(1,n))%*%glm_dropout2$coefficients)

f1_untreated=inv.logit(cbind(rep(1,n),baselines,rep(0,n))%*%glm_dropout1$coefficients)
f2_untreated=inv.logit(cbind(rep(1,n),exp(logtumourratios[1])*baselines,rep(0,n))%*%glm_dropout2$coefficients)

pdf_treated=dmvnorm(cbind(-means_treated[,1]+logtumourratios[1],-means_treated[,2]+logtumourratios[2]),
mean=c(0,0),sigma=matrix(c(Sigma[1,1],Sigma[1,2],Sigma[2,1],Sigma[2,2]),2,2))
pdf_untreated=dmvnorm(cbind(-means_untreated[,1]+logtumourratios[1],-means_untreated[,2]+logtumourratios[2]),
mean=c(0,0),sigma=matrix(c(Sigma[1,1],Sigma[1,2],Sigma[2,1],Sigma[2,2]),2,2))

return(mean((1-f1_treated)*(1-f2_treated)*pdf_treated)-mean((1-f1_untreated)*(1-f2_untreated)*pdf_untreated))
}


probabilityofsuccess=function(continuousmodel,dropoutmodel1,dropoutmodel2,baselines,dichotomisationthreshold)
{
#probabilityofsuccess integrates over ‘integrand’ to find the mean difference in probability of success
#arguments - 1) gls object representing the tumour shrinkage model; 2),3) the two glm objects modelling
#the probability of dropout between each visit; 4) the vector of baselines for all patients;
#5) the dichotomisation threshold (on the percentage scale) used to classify patients as successes or failures.

#get the vector of mean tumour shrinkage were all patients untreated, and if all were treated
means_treated=cbind(cbind(rep(1,length(baselines)),rep(1,length(baselines)),rep(0,length(baselines)),
baselines,rep(1,length(baselines)))%*%continuousmodel$coefficients,cbind(rep(1,length(baselines)),
rep(0,length(baselines)),rep(1,length(baselines)),baselines,rep(2,length(baselines)))%*%continuousmodel$coefficients)

means_untreated=cbind(cbind(rep(1,length(baselines)),rep(0,length(baselines)),rep(0,length(baselines)),
baselines,rep(1,length(baselines)))%*%continuousmodel$coefficients,cbind(rep(1,length(baselines)),
rep(0,length(baselines)),rep(0,length(baselines)),baselines,rep(2,length(baselines)))%*%continuousmodel$coefficients)

#find lower and upper points for integration:
maxmean1=max(c(means_treated[,1],means_untreated[,1]))
maxmean2=max(c(means_treated[,2],means_untreated[,2]))
minmean1=min(c(means_treated[,1],means_untreated[,1]))
minmean2=min(c(means_treated[,2],means_untreated[,2]))

#integrate

a=cuhre(2,1,integrand=integrand,means_treated=means_treated,means_untreated=means_untreated,
Sigma=getVarCov(continuousmodel),glm_dropout1=dropoutmodel1,glm_dropout2=dropoutmodel2,baselines=baselines,
lower=c(qnorm(1e-08,minmean1,sqrt(getVarCov(continuousmodel)[1,1])),qnorm(1e-08,minmean2,
sqrt(getVarCov(continuousmodel)[2,2]))),upper=c(qnorm(1-1e-08,maxmean1,sqrt(getVarCov(continuousmodel)[1,1])),
```

```
log(dichotomisationthreshold)),flags=list(verbose=0,final=1,pseudo.random=0,mersenne.seed=NULL))

return(a$value)
}


partialderivatives=function(continuousmodel,dropoutmodel1,dropoutmodel2,baselines,dichotomisationthreshold)
{
#finds numerical partial derivatives of probability of success w.r.t to parameters in the continuous model
#and both glm model value at current parameters

value=probabilityofsuccess(continuousmodel,dropoutmodel1,dropoutmodel2,baselines,dichotomisationthreshold)

partials=rep(0,10)

#partials w.r.t continuous parameters first:
for(i in 1:5)
{
continuousmodel_temp=continuousmodel
continuousmodel_temp$coefficients[i]=continuousmodel_temp$coefficients[i]+0.000001

value_temp=(probabilityofsuccess(continuousmodel_temp,dropoutmodel1,dropoutmodel2,baselines,
dichotomisationthreshold)-value)/0.000001

partials[i]=value_temp
}

#dropout model 1:

for(i in 1:3)
{
dropoutmodel1_temp=dropoutmodel1
dropoutmodel1_temp$coefficients[i]=dropoutmodel1_temp$coefficients[i]+0.000001

value_temp=(probabilityofsuccess(continuousmodel,dropoutmodel1_temp,dropoutmodel2,baselines,
dichotomisationthreshold)-value)/0.000001

partials[(i+5)]=value_temp
}
#dropout model 2:

for(i in 1:3)
{
dropoutmodel2_temp=dropoutmodel2
dropoutmodel2_temp$coefficients[i]=dropoutmodel2_temp$coefficients[i]+0.000001

value_temp=(probabilityofsuccess(continuousmodel,dropoutmodel1,dropoutmodel2_temp,baselines,
dichotomisationthreshold)-value)/0.000001
partials[i+8]=value_temp
}
#return the partial derivatives and the actual probability
return(c(partials,value))
}


#y is the log-tumour shrinkage for each individual at both time points;
```

```
#id indexes which individual each datapoint corresponds to
#X is the design matrix, with a column for the intercept; a column representing the treatment
#indicator at the first visit; a column representing the treatment indicator for the second
#visit; and a column representing the baseline tumour size of each individual
#time is a vector labelling which visit the observation comes from
#dropout1 is a vector of 1's and 0's which represent if an individual drops out
#due to death/tox between baseline and the interim
#dropout2 is as above, but 1 if the individual drops out after the interim; only individuals
#who made it past the interim are included in this vector.
#baseline is the vector of each individual's baseline tumour size;
#interim is the vector of each individual's interim tumour size, assuming they did not drop out
#treatment is the treatment indicator.

dropoutmodel1=glm(dropout1~baseline+treatment,family="binomial")
dropoutmodel2=glm(dropout2~interim+treatment[dropout1==0],family="binomial")

continuousmodel=gls(y~X[,-1]+time,correlation=corSymm(form=~1|id),weights=varIdent(form=~1|time),na.action=na.omit)

partials=partialderivatives(continuousmodel,dropoutmodel1,dropoutmodel2,baseline,0.7)

mean=partials[12]
partials=partials[1:11]

covariance=matrix(0,11,11)

covariance[1:5,1:5]=continuousmodel$varBeta
covariance[6:8,6:8]=summary(dropoutmodel1)$cov.unscaled
covariance[9:11,9:11]=summary(dropoutmodel2)$cov.unscaled

#delta method approximation of variance
variance=t(partials)%*%covariance%*%partials

#confidence interval for difference in mean probability of success
CI=c(mean-1.96*sqrt(variance),mean,mean+1.96*sqrt(variance))
```
